# Structural Characterization and Association of Ovine Dickkopf-1 Gene with Wool Production and Quality Traits in Chinese Merino

**DOI:** 10.3390/genes8120400

**Published:** 2017-12-20

**Authors:** Fang Mu, Enguang Rong, Yang Jing, Hua Yang, Guangwei Ma, Xiaohong Yan, Zhipeng Wang, Yumao Li, Hui Li, Ning Wang

**Affiliations:** 1 Key Laboratory of Chicken Genetics and Breeding at Ministry of Agriculture, Key Laboratory of Animal Genetics, Breeding and Reproduction at Education Department of Heilongjiang Province, Key Laboratory of Animal Cellular and Genetic Engineering of Heilongjiang Province, Harbin 150030, China; 18103631565@163.com (F.M.); reg3641024@163.com (E.R.); 18846084795@163.com (Y.J.); maguangwei77@163.com (G.M.); yanxiaohong@neau.edu.cn (X.Y.); wangzhipeng@neau.edu.cn (Z.W.); liyumao@neau.edu.cn (Y.L.); lihui@neau.edu.cn (H.L.); 2 Institute of Animal Husbandry and Veterinary, Xinjiang Academy of Agriculture and Reclamation Science, Shihezi 832000, China; yhxjcn@sina.com

**Keywords:** Chinese Merino, Dickkopf-1, genomic structure, single nucleotide polymorphism, wool quality traits

## Abstract

Dickkopf-1 (DKK1) is an inhibitor of canonical Wnt signaling pathway and regulates hair follicle morphogenesis and cycling. To investigate the potential involvement of *DKK1* in wool production and quality traits, we characterized the genomic structure of ovine *DKK1*, performed polymorphism detection and association analysis of ovine *DKK1* with wool production and quality traits in Chinese Merino. Our results showed that ovine *DKK1* consists of four exons and three introns, which encodes a protein of 262 amino acids. The coding sequence of ovine *DKK1* and its deduced amino acid sequence were highly conserved in mammals. Eleven single nucleotide polymorphisms (SNPs) were identified within the ovine *DKK1* genomic region. Gene-wide association analysis showed that SNP5 was significantly associated with mean fiber diameter (MFD) in the B (selected for long wool fiber and high-quality wool), PW (selected for high reproductive capacity, high clean wool yield and high-quality wool) and U (selected for long wool fiber with good uniformity, high wool yield and lower fiber diameter) strains (*p* < 4.55 × 10^−3^ = 0.05/11). Single Nucleotide Polymorphisms wide association analysis showed that SNP8 was significantly associated with MFD in A strain and fleece weight in A (selected for large body size), PM (selected for large body size, high reproductive capacity and high meat yield) and SF (selected for mean fiber diameter less than 18 μm and wool fiber length between 5 and 9 cm) strains (*p* < 0.05), SNP9 was significantly associated with curvature in B and U strains (*p* < 0.05) and SNP10 was significantly associated with coefficient of variation of fiber diameter in A, PW and PM strains and standard deviation of fiber diameter in A and PM strains (*p* < 0.05). The haplotypes derived from these 11 identified SNPs were significantly associated with MFD (*p* < 0.05). In conclusion, our results suggest that *DKK1* may be a major gene controlling wool production and quality traits, also the identified SNPs (SNPs5, 8, 9 and 10) might be used as potential molecular markers for improving sheep wool production and quality in sheep breeding.

## 1. Introduction

Wool is an important valuable economic product of Merino sheep and plays an important role in textile industry. Wool still is a vital source of income in sheep operation although the proportion of income derived from wool sales has decreased. In Xinjiang Uyghur Autonomous Region where Chinese Merino sheep are raised, wool generally accounts for 21% of gross flock income. The value of wool is determined by its intrinsic quality which includes a number of wool quality traits. From these traits, mean fiber diameter (MFD) is the most important wool characteristics when assessing value. The variation of mean fiber diameter, expressed as the standard deviation of the mean fiber diameter (FDSD) and coefficient of variation of the mean fiber diameter (CVFD), is also an important trait for wool processing. Less variation in fiber diameter is desirable to wool processor because of its better spinning quality. Wool production and quality traits are polygenic and several of genes have been identified to be associated with wool production and quality trait. For example, it has been shown that the Desmoglein 4 (*DSG4*) gene is associated with wool length and curvature [1], while the Beta-3 adrenergic receptor (*ADRB3*) gene is associated with wool mean staple strength and yield [2].

Dickkopf-1 (DKK1), a member of the dickkopf family, is a secreted canonical Wnt signaling inhibitor [3]. The canonical Wnt signaling pathway plays important roles in developmental processes, organogenesis, oncogenetic and self-renewal during tissue morphogenesis [4,5]. In the absence of the Wnt ligands, cytoplasmic β-catenin is phosphorylated and constantly degraded by the destruction complex formed by Axin, APC (WNT signaling pathway regulator), casein kinase 1 (CK1) and glycogen synthase kinase 3 beta (GSK-3β). In the presence of Wnt ligands, they bind to the Frizzled/LRP receptor complex at the cell surface. These receptors transduce a signal into the cells. As a consequence, the degradation of β-catenin is inhibited and β-catenin accumulates in the cytoplasm. The accumulated β-catenin travels to the nucleus to form complexes with the T cell factor/lymphoid enhancer binding factor (TCF/LEF) and activates Wnt target gene expression [6]. The canonical Wnt signaling regulates hair follicle morphogenesis [7], cycling [8] and hair follicle stem cell proliferation [9]. It has also been demonstrated that DKK1 regulates hair follicle density [10], terminal hair [11], hair follicle size [12] and hair follicle cycling [13,14] by inhibiting canonical Wnt signaling. The hair follicle size, density and cycling have an effect on the hair or wool length and fiber diameter [15,16,17]. Although it has been demonstrated that DKK1 regulates hair follicle morphogenesis and cycling, its effects on hair production and quality traits are unclear.

The aim of this work was to investigate the potential involvement of DKK1 in wool production and quality traits. We characterized the genomic structure of ovine *DKK1* and performed single nucleotide polymorphism screening and association analysis of the single nucleotide polymorphisms (SNPs) and haplotypes of *DKK1* with wool production and quality traits in Chinese Merino population. 

## 2. Materials and Methods

### 2.1. Animals and Trait Measurements

A total of 743 ewes from Chinese Merino breed population (Xinjiang Junken type) comprising 181 superfine wool sheep (SF), 134 prolific meat sheep (PM), 138 prolific wool sheep (PW), 151 A strain, 103 B strain and 36 U strain were genotyped and phenotyped in this study. These six Chinese Merino strains were selected for different purposes. The SF strain was selected for mean fiber diameter less than 18 μm and wool fiber length between 5 and 9 cm. The PM strain was selected for large body size, high reproductive capacity and high meat yield. Its wool fiber length ranged between 9.30 and 12.11 cm, while mean fiber diameter ranged between 20.72 and 23.65 μm. The PW strain was selected for high reproductive capacity, high clean wool yield and high-quality wool. The reproduction rate of PM and PW was 182.4%, which is more than 60% higher compared to SF. The A strain was selected for large body size, while B strain was selected for long wool fiber and high-quality wool. The U strain was selected for long wool fiber with good uniformity, high wool yield and lower fiber diameter. Its mean fiber diameter ranged between 15.58 μm and 20.79 μm [18]. All animals were fed ad libitum with grazing diet and maintained under the same conditions of environment, feeding and management. Procedures involving animals and their care were conducted in conformity with the guidelines of National Institutes of Health guidelines [19] (NIH Publication No. 85-23, revised 1996) and the Ministry of Science and Technology of the People’s Republic of China (Approval number:2006-398, 30 September 2006) and were approved by the Laboratory Animal Management Committee of the Northeast Agricultural University (Harbin, China) and Xinjiang Academy of Agriculture and Reclamation Science (Shihezi, China) (2014ZX08009-002 and 2009ZX08009-160B, 5 February 2010). 

Wool sample were collected from ewes before pregnancy, which occurred in May 2009. All ewes were aged from 1 to 12 years and shorn at the same year. According to the guidelines of the China Fiber Inspection Bureau (CFIB) and International Wool Textile Organization (IWTO), the wool production and quality traits (MFD, FDSD, CVFD, wool fiber length (WFL), curvature and fleece weight (FW)) were measured in this study. The MFD, FDSD and CVFD were measured using an instrument called OFDA2000 (BSC Electronics, Ardross, Australia) which is recognized by the IWTO test method (47 and 57). Wool fiber length was measured in centimeters and reflected the relaxed length of the staple under no tension on the spine, above the last rib. A crimp is defined as the distance from one peak to the next in the wool staple. Curvature was measured by using the OFDA2000 (BSC Electronics). Fleece weight and the total sheared fleece were weighed [20].

### 2.2. Genomic DNA Isolation

Ear notch samples were collected and genomic DNA was extracted using the standard phenol-chloroform method [21], then stored for genotyping. DNA concentration and purity were measured using the NanoDrop 2000 spectrophotometer (Thermo Scientific, Irvine, CA, USA). An absorbance 260/280 ratio between 1.8 and 2.0 AU and an absorbance 260/230 ratio between 2.0 and 2.2 AU represent a high-quality DNA sample. Genomic DNA integrity was determined by 1% agarose gel electrophoresis using the intercalating agent GelRed™ (Biotium, Fremont, CA, USA) 10,000× with the Bromophenol Blue as carrier, then visualized under ultraviolet light (UV) and photographed.

### 2.3. RNA Isolation and Reverse Transcription

The Liver, testis and kidney tissues of the six strains of Chinese Merino sheep were collected (*n* = 18, three for each strain). The total RNAs from these samples were isolated using TRIzol reagent (Invitrogen, Rockville, MD, USA) according to the manufacturer’s instructions. RNA concentration and purity were measured using the Nanodrop 2000 spectrophotometer (Thermo Scientific). Absorbance 260/280 ratio was used to assess the purity of the isolated total RNAs. The absorbance 260/280 ratio between 1.8 and 2.0 indicate good RNA purity. RNA integrity was determined by 1.2% formaldehyde denaturing gel electrophoresis. To eliminate genomic DNA (gDNA) contamination, all isolated RNAs were treated with RNase-free DNase I (Qiagen Inc., Hilden, Germany). Complementary DNA (cDNA) was synthesized from 1 μg of total RNA using Promega Improm-II reverse transcription System (Promega, Madison, WI, USA) following the manufacturer’s instructions.

### 2.4. PCR Amplification

The synthesized cDNA was used as a template to amplify the entire coding sequence of ovine *DKK1* by PCR using gene specific primer *DKK1*-F1 and *DKK1*-R1 (Table 1), which were designed according to the predicted cDNA sequence of ovine *DKK1* (GenBank accession No. XM_012138945.2). The PCR amplification of *DKK1* cDNA was performed in a 50 μL reaction volume containing 1 μL of Phanta Max Super-Fidelity DNA Polymerase (Vazyme Biotech Co., Ltd., Nanjing, China), 1 μL cDNA, 25 μL of 2× Phanta Max buffer, 1 μL of 10 mM dNTP Mix, 2 µL of forward primer (10 µM), 2 µL of reverse primer (10 µM) and 18 μL of RNase-free water. The cycling protocol was 95 °C for 3 min, 30 cycles of 95 °C for 15 s, 59 °C for 15 s, 72 °C for 1 min and a final extension at 72 °C for 5 min. The products were analyzed by 1% agarose gel electrophoresis.

To obtain the full-length ovine *DKK1* genomic sequence, a total of five primer pairs, were designed to cover the entire genomic region of ovine *DKK1* based on the caprine *DKK1* genomic sequence (GenBank accession No. GQ480837) and the bovine genome sequence from the assembly of chromosome 26 reported in Genbank (accession number NM_001205544) and the *Bos taurus* genome sequence (http://genome.ucsc.edu). The 3′-terminus of each amplified fragment overlapped with the 5′-terminus of its adjacent amplified fragment. The primer sequences are listed in Table 1. The PCR amplification of the *DKK1* genomic sequence was performed in a 50 μL reaction volume containing 1 μL of Phanta Max Super-Fidelity DNA Polymerase (Vazyme Biotech Co., Ltd.), 100 ng ovine gDNA, 25 μL of 2× Phanta Max buffer, 1 μL of 10 mM dNTP Mix, 2 µL of forward primer (10 µM), 2 µL of reverse primer (10 µM) and RNase-free water to final volume of 50 μL. The genomic PCR conditions included: 95 °C for 3 min followed by 35 cycles at 95 °C for 15 s and 46.9 °C (52.0, 56.2, 56.2, 58.6 °C) for 15 s, 72 °C for 2 min and a final extension at 72 °C for 5 min. The products were analyzed by 1% agarose gel electrophoresis.

### 2.5. Sequencing and Sequence Analysis

PCR products of *DKK1* cDNA and genomic fragments were purified using the Agarose Gel Extraction Kit according to the manufacturer’s instructions (TIANGEN, Beijing, China) and cloned into pGEM-T Easy Vector (Promega, Madison, WI, USA). The recombinant plasmids were extracted using PureLinkR Quick Plasmid Miniprep kit (Invitrogen) and sequenced by Invitrogen. The sequences were aligned using the Align X function of Vector NTI program (Informax, Rockville, MD, USA). The homologous *DKK1* mRNA sequences of 16 different animal species used in this study were obtained from National Center for Biotechnology Information (NCBI, https://www.ncbi.nlm.nih.gov/) database (Table S1). Homology analysis was performed using the Align Sequences Nucleotide BLAST utility at NCBI (https://blast.ncbi.nlm.nih.gov/Blast.cgi). Translation of the nucleotide sequences into the amino acids was performed using the DNAMAN program (Lynnon Corp., Quebec, Canada). Exon–intron boundaries were identified by alignment of the acquired Merino *DKK1* genomic DNA sequence (GenBank accession No. JQ348893.1) and the acquired Merino *DKK1* cDNA sequence using the Align Sequences Nucleotide BLAST at NCBI. The signal peptide was analyzed using SignalP 3.0 (http://www.cbs.dtu.dk/services/SignalP/). The conserved domains were analyzed using a conserved domain database (CDD) (http://www.ncbi.nlm.nih.gov/cdd/). Transcription factor binding sites (TFBS) were predicted using Mulan (https://mulan.dcode.org/). Core promoter was predicted using Promoter SCAN (http://www-bimas.cit.nih.gov/molbio/proscan/). The neighbor-joining phylogenetic tree was constructed using the Phydit program version 3.0 [22] based on genetic distances calculated with Kimura’s two-parameter method [23].

### 2.6. Identification of Polymorphisms of the Ovine DKK1

To screen for *DKK1* polymorphisms, a total of five primer pairs (DKK1-F2/R2-DKK1-F6/R6) were used to amplify DKK1 genomic region (Table 1) using the pooled gDNA as the template, which were from 60 Chinese Merino individuals as previously described [24]. The PCR products obtained from pooled genomic DNA sample were directly sequenced by Sanger sequencing and a SNP was ascertained by the presence of a double peak at the level of a single base in the chromatograms of sequencing of pooled PCR products [25].

### 2.7. Genotyping of the Ovine DKK1 

A multiplexed SNP single base extension (SBE) assay was used for SNP genotyping. It was performed using a 384 well plate format on the Sequenom Mass ARRAY platform (Bioyong Technologies Inc., Beijing, China). The Mass ARRAY Assay Design 3.1 software (Sequenom, San Diego, CA, USA) was used to design amplification and allele-specific extension primers. The raw data files generated by Mass Array (Sequenom) were analyzed for the intensity peaks of calibrant to ascertain the quality of the data as previous described [26,27]. An overall call rate of greater than 95% was maintained. For every 96 samples (a quadrant of the Sequenom chip), four samples were duplicated and the call rates were checked for concordance. The calls in the negative control (no DNA) were also monitored in all the runs. The reproducibility of this study was 100%. 

### 2.8. Statistical Analysis

Data are summarized as mean values for each parameter measured in each group. Correlation analysis between wool production and quality traits was subjected to the Pearson procedure of SPSS 22.0 (IBM, Armonk, NY, USA). Genotype and allelic frequencies at each SNP site were calculated, with the allele frequencies in subjects for each SNP evaluated for deviation from Hardy–Weinberg equilibrium and differences between groups using the χ^2^ test using Statistical Analysis System (SAS Inst. Inc., Cary, NC, USA). 

Haplotypes for each individual were obtained in SAS/GENETICS using the PROC HAPLOTYPE procedure. This procedure uses the Expectation Maximization (EM) algorithm to generate maximum likelihood estimates of the haplotype frequencies. Before analyzing the association between the identified SNPs and wool production and quality traits, we performed the data preprocessing: if the number of one genotype was fewer than 5% × the total number of samples, we removed the data for this genotype.

According to the characteristics of the Chinese Merino population, associations of *DKK1* SNPs, haplotypes or allele substitution effect with wool production and quality traits were analyzed using mixed linear model procedure in SAS (SAS Inst. Inc.). The model was as below:

*Y* = *μ* + *A* + *S* + *L* + *G* [*L*] + *e*

where *Y* is the phenotypic value for each individual, *μ* is the population mean, *A* is the continuous effect of the age, *S* is the random effect of the sire, *L* is the fixed effect of the line, *G* [*L*] is the effect of genotype nested within line and e is the random error; Data were subjected to the John’s Macintosh Program 7.0 (JMP, SAS Inst. Inc.) which was used to examine the correlation between genotypes and haplotypes and continuous traits (MFD, FDSD, CVFD, WFL, curvature and FW) and to evaluate the least squares means. The genetic and phenotypic correlations between the wool production and quality traits were estimated using ASREML software [28], with line treated as a fixed effect. The bivariate model was used to calculate the genetic and phenotypic correlations.

The standard errors (SE) of correlations between genotypes and wool production and quality traits were approximated as described in the study of Falconer and Mackay [29]. The genetic model used for parameter estimations is described as follows:

*y* = *Xβ* + *Zu* + *e*

in which *y* is an *n*-dimensional vector of observed values for the traits, *X* is an *n* × *p* matrix of the fixed effects, *β* is a *p*-dimensional vector of the fixed effects, *Z* is an *n* × *q* matrix of the random effects, *u* is a *q*-dimensional vector of the random genetic effects and *e* is an *n*-dimensional vector of the random residual effects.

The random effects *u* and *e* were assumed to follow the normal distributions with mean 0, that is, Expectation [*y*] = *Xβ*. The variances of *u* and *e* were assumed to be *Var(u)* = *Ag* and *Var(e)* = Ir, respectively, in which *A* is the numerator relationship matrix of all animals in the sire file, *g* is the additive genetic variance for the single-variate and the additive genetic variance–covariance matrix between traits for the bivariate model analysis, *I* is the identity matrix of order equal to the number of animals with phenotypes and *r* is the residual variance for the single-variate and the variance–covariance matrix between residuals on the same animal when performing the bivariate model analysis, where residual covariance equal to 0 [30]. Significance was evaluated based on an SNP-wide and gene-wide type I error rate of 0.05. The values were considered significant at *p* < 0.05 based on SNP-wide and threshold gene-wide which is *p* < 4.55 × 10^−3^ = 0.05/11 using Bonferroni correction.

## 3. Results

### 3.1. Structural Characterization of Ovine DKK1 

**Figure 1:**
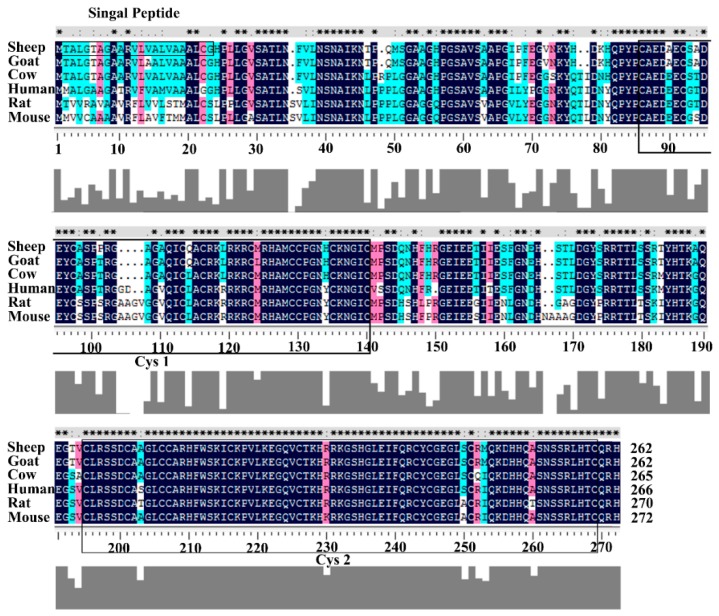
Alignment of the deduced amino acid sequences and major domains of sheep and other mammalian Dickkopf-1 (DKK1) proteins. * (asterisk), : (colon) and . (dot) mean identical amino acid residues, similar alternate amino acid residues and dissimilar alternate amino acid residues, respectively. Cys1 and Cys2 show N-terminal cysteine-rich domain and C-terminal cysteine-rich domain. Darker gray indicates greater homology; the degree of homology is indicated by shading.

**Figure 2:**
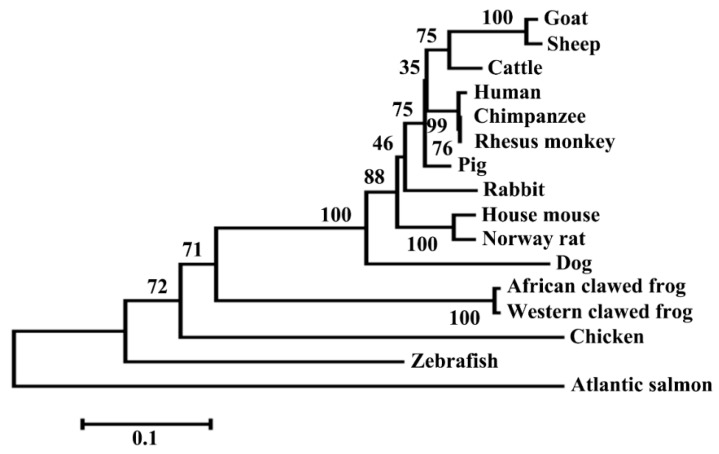
Phylogenetic analysis based on amino acid sequences of DKK1 in various animal species. The phylogenetic rooted tree was inferred using the same units as those of the evolutionary distance with branch lengths to scale. The Information for DKK1 amino acid sequences in 16 different animal species are listed in Table S1.

To investigate the potential involvement of the *DKK1* in wool production and quality traits in Chinese Merino sheep, we first determined the full-length coding sequence and genomic structure of ovine *DKK1*. The cDNA sequence of ovine *DKK1* was amplified by Real Time PCR (RT-PCR) from the pooled total RNA of liver, testis and kidney using the primer pair (*DKK1*-F1 and *DKK1*-R1) (Table 1). Sequencing results showed that the amplified *DKK1* cDNA fragment is 955 bp long. Further sequence analysis showed that this fragment contained the full-length coding region sequence (789 bp) of ovine *DKK1* which encodes a protein of 262 amino acids. The entire coding sequence of *DKK1* of Chinese Merino shared 100% nucleotide sequence identity with the recently published Texel sheep *DKK1* mRNA sequence (GenBank accession No. XM_012138945.2). The coding sequence of the ovine *DKK1* showed 98.10%, 93.61% and 83.96% nucleotide identity with those of caprine, bovine and human *DKK1*, respectively. The *DKK1* deduced amino acid sequence alignment and protein domain analysis are shown in Figure 1. Analysis of the deduced amino acid sequence of ovine *DKK1* using SignalP3.0 (http://www.cbs.dtu.dk/services/SignalP) identified a potential signal peptide sequence at its amino terminus positions 1–23, consistent with its role as a secreted protein. The conserved domain analysis showed that ovine DKK1 protein contained an N-terminal cysteine-rich domain and a C-terminal cysteine-rich domain at amino acid positions 87–136 and 188–252 respectively. These two cysteine-rich domains were conserved in six different animal species analyzed. The deduced ovine DKK1 protein showed high sequence similarity to caprine (98.85%), bovine (92.08%) and human (83.90%) DKK1 proteins (Table S2). The phylogenetic analysis based on amino acid sequence of DKK1 protein showed that that Merino sheep was closely clustered with goat and cattle while Human, Chimpanzee and Rhesus monkey formed another closely related group. In contrast, the Atlantic salmon was a distinct group compared with other species (Figure 2). 

The genomic sequence of ovine *DKK1* was amplified by PCR from the gDNA of Chinese Merino sheep using five different pairs of primer (Table 1) covering the entire *DKK1* genomic sequence. The five primer pairs generated five different sized PCR products: 1771 bp (chr22:6668693-6670458), 1107 bp (chr22:6667603-6668709), 2100 bp (chr22:6665566-6667623), 407 bp (chr22:6665250-6665656) and 1966 bp (chr22:6663304-6665269. All PCR products were sequenced and assembled into the ovine *DKK1* genomic sequence. The acquired ovine *DKK1* genomic sequence was 7326 bp in length and has been deposited in GenBank (accession No. JQ348893). 

**Figure 3:**
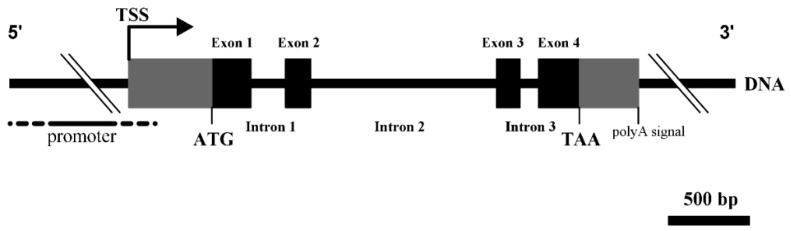
The genomic structure of the ovine *DKK1*. Exons: boxes. Introns: lines. The initiation codon (ATG) and the transcription starting site (TSS) are located in exon 1 and the stop codon (TAA) is located in exon 4.

Alignment of the acquired Merino *DKK1* genomic sequence and its cDNA sequence revealed that Merino *DKK1* is composed of three introns and four exons (Figure 3). The consensus sequences at the exon/intron boundaries were identified and all the boundaries conformed to the GT–AG rule. A putative polyA signal sequence (AATAAA), was found at 361 to 366 bp downstream of the termination codon (TAA). Sequence alignment analysis showed that our acquired Merino *DKK1* genomic sequence displayed high similarity to the recently published Texel sheep *DKK1* genomic sequence (GenBank accession No. NC_019479) (97.59%) and bovine *DKK1* genomic sequence (94.38%). Comparison of the Texel sheep and Merino *DKK1* genomic sequence showed that Merino *DKK1* genomic region had a 5 bp insertion in its 5′ flanking region and a 105 bp deletion in 3′ flanking region. Comparison of the ovine and caprine *DKK1* genomic structure showed that ovine and caprine *DKK1* shared the same numbers and sizes of exons and introns and their exon and intron sequences were highly similar to each other. The *DKK1* genomic sequence from the start codon ATG to the terminal codon TAA was 95.77% similar between sheep and goat.

### 3.2. Bioinformatics Analysis of Ovine DKK1 Promoter

Alignment of the acquired ovine *DKK1* genomic sequence (GenBank accession No. JQ348893) and its cDNA sequence (GenBank accession No. XM_012138945.2) identified a 2752 bp genomic sequence upstream of the initiation start codon (ATG) of ovine *DKK1*. To gain insight into the transcriptional regulation of ovine *DKK1*, we analyzed this 2752 bp upstream genomic sequence by using promoter prediction software Promoter SCAN (http://www-bimas.cit.nih.gov/molbio/proscan/) and Mulan (https://mulan.dcode.org/). The Promoter SCAN analysis showed that the transcriptional initiation site of ovine *DKK1* was at an A residue 515 bp (−515 bp) upstream of the initiation start codon (ATG), its promoter contained a canonical TATA box and a GC box at nucleotide −23 to −30 and −108 to −95 relative to its predicted transcriptional initiation site as revealed by using Promoter SCAN and Mulan software respectively. The TATA box and GC box were conserved in ovine, bovine and caprine *DKK1* promoters. Sequence alignment of the 2752 bp genomic sequence and caprine genomic sequence (GenBank accession No. GQ480837) identified a 251 bp sequence containing 94.27% nucleotide identity. This conserved 251 bp region was located at nucleotides −263 to −13 relative to the predicted transcriptional initiation site of ovine *DKK1*. A number of binding sites for transcription factors including RAR-related orphan receptor A isoform 1 (RORA1), signal transducers and activators of transcription 1 (STAT1), OCT4 (also called POU domain, class 5, transcription factor-1) were predicted at this conserved region, further analysis showed that RORA1, STAT1 and OCT4 binding sites were conserved among sheep, goat, cattle, pig, chimpanzee, human, rhesus monkey, rabbit, house mouse and Norway rat *DKKl* promoters. In addition, Mulan program predicted a conserved p53 binding sites in sheep, cattle, rabbit and mouse *DKK1* promoters; and one conserved TCF/LEF binding site in sheep, cattle, pig, human, rhesus monkey, rabbit, house mouse and Norway rat *DKK1* promoters; and nine SP1 in sheep and cattle *DKK1* promoters. The high degree of conservation of these transcription factor binding sites in *DKK1* promoters between these different animals indicates that the transcriptional regulation of *DKK1* may be similar in these animals.

### 3.3. Identification of SNP in DKK1

By PCR and sequencing, we detected SNPs in the *DKK1* genomic region from the pooled gDNA sample. A total of 11 SNPs were identified and named as SNPs1 to 11. The detailed SNP information is summarized in Table 2. Of these 11 SNPs, SNPs1 to 5 were located in intron 2, SNP6 in intron 3, SNP7 in exon 4 which is a silent mutation, SNPs8 and 9 in the 3' UTR and SNPs10 and 11 in the 3′ flanking region of ovine *DKK1* (Table 2).

### 3.4. Allele, Genotype and Haplotype Frequencies of Ovine DKK1 

A total of 743 individuals of the six Chinese Merino strains (SF, PW, PM, A, B and U) were genotyped for the 11 identified SNPs using the SBE assay. For SNPs1 to 8, the frequency of the alleles (G of SNP1, C of SNP2, D of SNP3, G of SNP4, G of SNP5, G of SNP6, G of SNP7 and T of SNP8) is predominantly higher than that of the alternative alleles (A of SNP1, A of SNP2, I of SNP3, C of SNP4, T of SNP5, A of SNP6, A of SNP7 and C of SNP8) in all 6 tested Chinese Merino strains (Table S3). The minor allele frequency of these 11 identified SNPs varied from 15.9% to 49.0% and the SNP1 and SNPs6 to 9 were in Hardy-Weinberg equilibrium (Table 2; *p* > 0.05). The frequencies of the alleles and genotypes are shown in Table S3. The χ^2^ test results showed that the allele frequencies for these 11 identified SNPs were significantly different among the six strains studied (*p* < 0.01). Among three genotypes of these 11 identified SNPs in six Chinese Merino sheep strains, only the heterozygous genotype TC of SNP11 was not identified in the U strain. There were 12 haplotypes based on the identified SNPs in all tested individuals. The haplotype frequencies of Ovine *DKK1* differed among the Merino strains tested (Table S4).

### 3.5. Phenotypic and Genetic Correlations of the Wool Production and Quality Traits

Phenotypic and Genetic correlation coefficients were calculated for each tested trait. The results showed that in our studied population, there were moderate negative phenotypic correlations between curvature and MFD and FDSD (−0.41 for both) (Table S5; *p* < 0.05) and low negative phenotypic correlations between curvature and CVFD, WFL (−0.21 and −0.20, respectively) (Table S5). There was high positive phenotypic correlation between CVFD and FDSD (0.78), moderate positive phenotypic correlation between MFD and FDSD (0.57) (Table S5; *p* < 0.05) and low positive phenotypic correlation between WFL and FW (0.27) (Table S5; *p* < 0.05).

Genetic correlations between the traits ranged from −0.79 for curvature-MFD to 0.83 for MFD-FDSD (Table S5). There were high negative genetic correlations between curvature and MFD and FDSD (−0.79 for both) and moderate negative genetic correlation between curvature and CVFD (−0.43) (Table S5). There were high positive genetic correlations between MFD and FDSD (0.83), between WFL and FW (0.70) and between CVFD and FDSD (0.73) (Table S5; *p* < 0.01) and low positive genetic correlations between CVFD and MFD, FL (0.27 for both). 

### 3.6. Association of the Identified SNPs with Wool Production and Quality in Sheep

Association analysis using JMP 7.0 (SAS Inst. Inc.) showed that, of these 11 identified SNPs, SNP5 was significantly associated with MFD in B, PW and U strains (Table 3; *p* < 4.55 × 10^−3^ = 0.05/11). The Genotype GT of SNP5 was significantly associated with lower MFD than genotype GG in B and U strains and the Genotype GG of SNP5 was significantly associated with lower MFD than genotype GT in PW strains. (Table 3; *p* < 4.55 × 10^−3^ = 0.05/11). SNP-wide association analysis showed that SNP8 was markedly associated with MFD in A strain and it was also associated with FW in A, PM and SF strains (Table 3; *p* < 0.05). The Genotype CC of SNP8 was significantly associated with lower MFD and higher FW compared to genotypes TC and TT in A strain (Table 3; *p* < 0.05). The Genotype CC of SNP8 was significantly associated with higher FW than genotype TT in PM strain and the Genotype TT of SNP 8 was significantly associated with higher FW than genotype TC in SF strain. SNP9 was significantly associated with curvature in B and U strains (Table 3; *p* < 0.05). The Genotype TC of SNP9 was significantly associated with lower curvature than genotype CC in B strain; and the Genotype TT of SNP9 was significantly associated with lower curvature than genotype CC in U strain. SNP10 was significantly associated with CVFD in A, PW and PM strains and FDSD in A and PM strains (Table 3; *p* < 0.05). The Genotype TT of SNP10 was significantly associated with lower CVFD than genotype CC in A and PW strains and the Genotype TC of SNP10 was significantly associated with lower CVFD than genotype CC in PM strain. In contrast, SNPs1 to 4, 6, 7, 11 were not found to be associated with the wool production and quality traits in the tested Chinese Merino (Table 3; *p* > 0.05). 

### 3.7. Association of DKK1 Haplotypes with Wool Quality Traits

We also performed the haplotype association analysis using the same model used for the SNP association analysis. There was a total of 12 haplotypes based on the identified SNPs in all tested individuals. Among these haplotypes, haplotype H5 (27.2%) had the highest proportion. The haplotype association analysis results are summarized in Table 4. Sheep with haplotype H2 had a significantly lower MFD than the sheep with haplotype H5 in B strain. Sheep with haplotype H1 had a significantly lower MFD than the sheep with either haplotype H2 in PW strain or haplotype H5 in PM strain (Table 4; *p* < 0.05).

## 4. Discussion

In the present study, we characterized the full-length coding sequence and genomic structure of ovine *DKK1* and identified a total of 11 SNPs. The association analysis showed that *DKK1* polymorphisms were associated with MFD, FDSD, CVFD, FW and curvature in the tested population (*p* < 0.05).

The ovine *DKK1* genomic structure was found to be identical to that of another mammalian *DKK1*. The nucleotide and amino acid sequence analysis revealed that *DKK1* gene was conserved in mammals. Protein domain analysis showed that the C-terminal cysteine-rich region was conserved in mammalian DKK1 homologs, suggesting that the conserved C-terminal cysteine-rich region is essential for the function of DKK1. It has been reported that the C-terminal cysteine-rich region of *DKK1* is involved in binding to low-density lipoprotein receptor-related proteins (LRPs), which act as Wnt coreceptors and inhibits Wnt signaling [31]. 

The promoter analysis revealed that a GC box and a canonical TATA box were present upstream of ovine *DKK1*, suggesting ovine *DKK1* promoter is a classical promoter. In addition, we observed multiple conserved transcription factor binding sites (RORA1, STAT1, POU5F1, TCF/LEF1, p53 and SP1) in the ovine *DKK1* promoter region. Consistently, it has been reported that TCF/LEF-1 [32], p53 [33] and SP1 [34] transcriptionally regulate human *DKK1*. Determining whether these transcription factors directly regulate ovine *DKK1* would lead to a better understanding of the role of ovine *DKK1* in hair follicle morphogenesis, cycling and wool production.

In the present study, a total of 11 SNPs were identified in ovine DKK1 genomic region. From these 11 identified SNPs, six SNPs (SNPs2 to 5, 10, 11) were not in the Hardy-Weinberg equilibrium, which may be explained by the following reasons: (1) the alleles may be the predominant alleles during genetic evolution, thus being more conserved and more common than other alleles in this population [35]; (2) The number of sheep examined in each strain was not large enough that genetic drift makes a significant force [36]; (3) The alleles may be tightly linked with an advantageous allele; (4) The economically favorable traits were artificially selected [35].

The positive genetic correlations of MFD with WFL were consistent with the previous studies [37,38], although the genetic correlations were lower than those reported in the previous Merino studies. The previous Merino studies showed that the average genetic correlations of MFD-WFL were 0.19 and 0.29 [33,34]. In Targhee sheep, the genetic correlation of MFD-WFL was 0.30 [39]. The genetic correlation of MFD–FDSD (0.83) in our tested population was higher than that reported by Safari et al. in Merino study [40]. The genetic correlation between FDSD and FDCV (0.73) in this study agreed with the estimated value of 0.76 by Safari et al. [33] but the genetic correlation between MFD and FDCV (0.27) disagreed with the estimate value of −0.16 by Safari et al. [37]. This discrepancy may be due to the differences in sheep age and gender, genetic background and environment.

Our association analysis showed SNPs (SNPs 5, 8, 9 and 10) were significantly associated with wool quality traits in several Chinese Merino strains. The improvement of wool production and quality traits such as MFD, FDSD, CVFD, is one of the important goals in sheep breeding programs. We presume that the beneficial alleles of these four identified SNPs might be used for genetic improvement of wool quality traits in Chinese Merino population, which need to be verified in large sheep populations. Wnt pathway plays an important role in hair follicle morphogenesis and cycling [41,42]. DKK1 has been shown to be associated with hair follicle development [14,43]. Wool is the product of hair follicles, the hair follicle size, cycle and density affect hair or wool length, diameter, etc. [15,16,17]. Consistent with this view, our association analysis indicated that four *DKK1* SNPs (SNPs5, 8, 9 and 10) were associated with MFD, curvature, FDSD, CVFD and FW in several of our tested strains. 

From the four *DKK1* SNPs (SNPs5, 8, 9 and 10) associated with wool quality traits, SNP5 was located in intron 2, SNP8 and SNP9 were located in the DKK1 3′ UTR, while SNP10 was located in the 3′ flanking region. It has been demonstrated that some SNPs, which are located in introns, 3′ UTR and flanking regions, can affect gene expression [44,45,46,47,48,49,50,51]. We cannot exclude the possibility that these four identified SNPs (SNPs5, 8, 9 and 10) are functional and affect *DKK1* expression, causing changes in wool production and quality traits. It is worthwhile to further explore whether these SNPs affect *DKK1* expression in the future. 

## 5. Conclusions

In the present study, we cloned *DKK1* genomic and coding sequences of Merino sheep, an old and influential wool sheep which produce the finest and softest wool. Our results added new insight into ovine *DKK1* structure and the identified SNPs might be used as genetic molecular marker for genetic improvement of wool sheep.

## Figures and Tables

**Table 1:** List of PCR primers used in the study.

Primer Name	Coverage	Sequence (5′-3′)	Size (bp)	Annealing Temperature (°C)
*DKK1*-F1		GCAAAGCGACACTCCTCC	955	59.0
*DKK1*-R1		GCTCTTACACCCCAGATTTTCGG		
*DKK1*-F2	chr22:6668693-6670458	ATGAAACCGAACTCTTGACTTA	1771	58.6
*DKK1*-R2		AACTTGATTAGGCAGACACG		
*DKK1*-F3	chr22:6667603-6668709	GTCTGCYTAATCAAGTTCATCTAC	1107	56.2
*DKK1*-R3		GGTTCTTGATV^4^GCGTTGGAAT		
*DKK1*-F4	chr22:6665566-6667623	ATTCCAACGCB^1^ATCAAGAACC	2100	56.2
*DKK1*-R4		CAGR^2^CCTTCY^3^CCRCAGTAACA		
*DKK1*-F5	chr22:6665250-6665656	TCTCAAGGAAGGTCAAGTGT	407	52.0
*DKK1*-R5		GGTTGCATTTACAGGTAAGT		
*DKK1*-F6	chr22:6663304-6665269	ACTTACCTGTAAATGCAACC	1966	46.9
*DKK1*-R6		TTATCCTACAACTATATCAGCAC		

F: forward primer; R: reverse primer. Degenerate bases: B^1^, C/G/T; R^2^, A/G; Y^3^, C/T; V^4^, A/C/G.

**Table 2:** Identification and nomenclature of *Dickkopf-1 (DKK1)* Single Nucleotide Polymorphisms (SNPs).

SNP ID	Location	db SNP rs # ID ^1^	Alleles	Nomenclature ^2^	MAF ^3^	HW P ^4^
SNP1	Intron 2	rs424404921	G > A	g.909 G > A	0.217	0.0546
SNP2	Intron 2	rs411273297	C > A	g.927 C > A	0.217	0.0223
SNP3	Intron 2	rs592810220	Del CTG	g.1049 Del CTG	0.235	2.9 × 10^−37^
SNP4	Intron 2	rs409382156	G > C	g.1147 G > C	0.216	0.0070
SNP5	Intron 2	rs419857384	G > T	g.1321 G > T	0.159	1.2 × 10^−10^
SNP6	Intron 3	rs419902277	G > A	g.2018 G > A	0.385	0.4416
SNP7	exon 4	rs401363941	G > A	g.2062 G > A	0.327	0.948
SNP8	3′ UTR	rs428450289	T > C	g.3093 T > C	0.217	0.2552
SNP9	3′ UTR	rs415015281	C > T	g.3329 C > T	0.490	0.4864
SNP10	3′ Flanking Region	novel	T > C	g.4123 T > C	0.355	1.2 × 10^−19^
SNP11	3′ Flanking Region	novel	T > C	g.4443 T > C	0.471	1.3 × 10^−139^

^1^ db, database; rs #, reference SNP #; ^2^ Nomenclature, according to SNP position on the obtained *DKK1* DNA sequence (GenBank accession No. JQ348893); ^3^ MAF, minor allele frequency; ^4^ HW, Hardy–Weinberg test.

**Table 3:** Effects of *DKK1* genotypes on wool production and quality traits (least square means ± SE) ^1^.

Lines	SNP5		SNP8			SNP9		SNP10		
	Genotype	MFD (μm)	Genotype	MFD (μm)	FW (kg)	Genotype	Curvature (/2.5 cm)	Genotype	FDSD (μm)	CVFD
**A**	GG	21.08 ± 0.380 ^c^	CC	18.33 ± 1.362 ^d,e,f^	5.45 ± 0.515 ^a,c^	CC	11.56 ± 0.487 ^b,c,d,e^	CC	4.56 ± 0.150 ^a,b^	21.41 ± 0.608 ^a^
	GT	20.72 ± 0.522 ^c,d,e^	TC	22.31 ± 0.531 ^a^	4.97 ± 0.191 ^a,b^	TC	11.11 ± 0.514 ^e,f^	TC	4.30 ± 0.144 ^a,b,c,d^	20.62 ± 0.585 ^a,b,c^
	TT	21.12 ± 0.823 ^b,c,d,e^	TT	20.36 ± 0.463 ^c,d,e^	4.44 ± 0.185 ^c,d,e^	TT	11.52 ± 0.681 ^b,c,d,e,f^	TT	4.09 ± 0.247 ^c,d,e^	19.56 ± 1.002 ^b,c,d,e,f^
**B**	GG	21.54 ± 0.471 ^b,c^	CC	NE	NE	CC	13.50 ± 0.954 ^a,b^	CC	4.07 ± 0.193 ^c,d,e,g^	19.41 ± 0.784 ^b,c,d,e,f^
	GT	19.08 ± 0.817 ^e,f^	TC	19.78 ± 0.946 ^c,d,e,f^	4.95 ± 0.371 ^a,b,c,d^	TC	11.06 ± 0.639 ^c,e,f^	TC	4.12 ± 0.192 ^b,c,d,e^	20.03 ± 0.778 ^a,b,c,d,e^
	TT	19.89 ± 1.234 ^c,d,e,f^	TT	20.90 ± 0.489 ^b,c,d^	4.95 ± 0.186 ^a,b^	TT	13.56 ± 1.022 ^a,b,c^	TT	4.00 ± 0.247 ^b,c,d,e,f,g^	20.11 ± 1.002 ^a,b,c,d,e,f^
**PW**	GG	21.05 ± 0.454 ^c,d^	CC	22.59 ± 1.284 ^a,b,c^	4.58 ± 0.489 ^a,b,c,d,e,f^	CC	10.45 ± 0.765 ^e,f^	CC	4.64 ± 0.179 ^a^	20.76 ± 0.725 ^a,b,c,d^
	GT	22.86 ± 0.693 ^a,b^	TC	22.09 ± 0.631 ^a,b^	4.64 ± 0.250 ^a,b,c,d,e^	TC	11.35 ± 0.409 ^d,e,f^	TC	4.27 ± 0.157 ^a,b,c,d^	19.33 ± 0.637 ^c,d,e,f^
	TT	NE	TT	21.29 ± 0.514 ^a,b,c^	4.56 ± 0.196 ^a,b,c,d,e^	TT	11.61 ± 0.856 ^b,c,d,e,f^	TT	4.10 ± 0.268 ^a,b,c,d,e,f,g^	18.04 ± 1.086 ^e,f^
**PM**	GG	20.74 ± 0.504 ^c,d,e^	CC	20.73 ± 1.047 ^a,b,c,d,e,f^	4.19 ± 0.400 ^a,b,c,d,e,f^	CC	11.98 ± 0.687 ^b,c,d,e^	CC	4.41 ± 0.118 ^a,b,c^	21.09 ± 0.480 ^a,b^
	GT	19.73 ± 1.373 ^c,d,e,f^	TC	21.56 ± 0.655 ^a,b,c^	3.67 ± 0.252 ^f,g^	TC	10.97 ± 0.564 ^e,f^	TC	3.73 ± 0.276 ^d,e,f,g^	17.20 ± 1.120 ^f^
	TT	NE	TT	21.03 ± 0.482 ^a,b,c,d^	3.29 ± 0.191 ^g^	TT	12.17 ± 0.505 ^b,c,d,e^	TT	NE	NE
**U**	GG	23.74 ± 0.701 ^a^	CC	NE	NE	CC	8.88 ± 1.198 ^f^	CC	NE	NE
	GT	19.86 ± 1.307 ^c,d,e,f^	TC	22.06 ± 0.744 ^a,b,c^	4.17 ± 0.283 ^d,e,f^	TC	14.62 ± 1.640 ^a,b,d^	TC	NE	NE
	TT	NE	TT	20.21 ± 1.070 ^a,b,c,d,e,f^	4.10 ± 0.407 ^b,d,e,f,g^	TT	17.15 ± 1.771 ^a^	TT	NE	NE
**SF**	GG	18.79 ± 0.297 ^f^	CC	NE	NE	CC	15.44 ± 0.600 ^a^	CC	3.61 ± 0.129 ^f^	19.04 ± 0.522 ^d,e,f^
	GT	19.86 ± 1.307 ^c,d,e,f^	TC	19.24 ± 0.494 ^e,f^	4.07 ± 0.198 ^e,f^	TC	15.32 ± 0.434 ^a^	TC	3.75 ± 0.159 ^e,f,g^	19.45 ± 0.644 ^c,d,e,f^
	TT	20.09 ± 1.275 ^b,c,d,e,f^	TT	18.81 ± 0.359 ^f^	4.66 ± 0.139 ^a,b,c,d^	TT	14.74 ± 0.523 ^a^	TT	3.59 ± 0.168 ^f,g^	19.37 ± 0.682 ^c,d,e,f^
*p* value		0.0037 ^2^		0.0139	0.0456		0.0186		0.0363	0.0053

The complete trait data are only included for the traits associated with the identified SNPs; ^1^ Least square means within columns that do not share a lower-case superscript letter (a, b, c, d, e, f, g) are different, *p* < 0.05; MFD, means mean fiber diameter; FW, fleece weight; FDSD, standard deviation of the mean fiber diameter; CVFD, coefficient of variation of the mean fiber diameter; ^2^ *p* value was evaluated based on threshold gene-wide (*p* < 4.55 × 10^−3^ = 0.05/11) using Bonferroni correction; NE stands for not estimable.

**Table 4:** The influence of *DKK1* haplotypes on several wool quality traits.

Lines	Haplotype	MFD (μm)
**A**	H1: AAICGAGCCCC	NE
	H2: AAICTAATCTT	20.97 ± 0.628 ^a,b,c,d,e,g^
	H3: GCDGGAATCTT	22.43 ± 0.739 ^a,b^
	H4: GCDGGGGCCCC	NE
	H5: GCDGGGGTTCC	21.43 ± 0.670 ^a,b,c,d,e^
**B**	H1: AAICGAGCCCC	NE
	H2: AAICTAATCTT	18.43 ± 0.811 ^f^
	H3: GCDGGAATCTT	19.68 ± 0.654 ^d,e,f,g^
	H4: GCDGGGGCCCC	18.58 ± 1.369 ^d,e,f,g^
	H5: GCDGGGGTTCC	21.30 ± 0.881 ^a,b,c,d,e^
**PW**	H1: AAICGAGCCCC	20.10 ± 1.024 ^b,d,e,f,g^
	H2: AAICTAATCTT	23.21 ± 1.363 ^a,c^
	H3: GCDGGAATCTT	21.45 ± 0.627 ^a,b,c,d^
	H4: GCDGGGGCCCC	20.44 ± 1.624 ^a,b,c,d,e,f,g^
	H5: GCDGGGGTTCC	20.41 ± 0.811 ^a,b,c,d,e,f,g^
**PM**	H1: AAICGAGCCCC	18.43 ± 1.106 ^f,g^
	H2: AAICTAATCTT	21.05 ± 1.380 ^a,b,c,d,e,f,g^
	H3: GCDGGAATCTT	20.34 ± 0.692 ^c,d,e,f,g^
	H4: GCDGGGGCCCC	20.18 ± 0.678 ^d,e,f,g^
	H5: GCDGGGGTTCC	21.09 ± 0.679 ^a,b,c,d,e^
**U**	H1: AAICGAGCCCC	NE
	H2: AAICTAATCTT	NE
	H3: GCDGGAATCTT	NE
	H4: GCDGGGGCCCC	NE
	H5: GCDGGGGTTCC	NE
**SF**	H1: AAICGAGCCCC	19.81 ± 0.722 ^d,e,f,g^
	H2: AAICTAATCTT	20.16 ± 0.519 ^d,e,f,g^
	H3: GCDGGAATCTT	18.81 ± 0.538 ^f^
	H4: GCDGGGGCCCC	NE
	H5: GCDGGGGTTCC	19.89 ± 0.424 ^e,f,g^
*p* value		0.014

Before the association analysis of the identified haplotypes with wool quality traits. That is, if the number of one haplotype was fewer than 5% × the total number of samples, we removed the data for this haplotype. Only traits associated with the identified haplotypes are presented; ^a,b,c,d,e,f,g^ Mean within a column with no common superscript are different (*p* < 0.05); NE stands for not estimable.

## Supplementary Materials

The following are available online at www.mdpi.com/2073-4425/8/12/400/s1. Table S1: Information for *DKK1* from 16 different animal species, Table S2: The similarities of the deduced amino acids sequence of DKK1 protein in 16 different animal species, Table S3: Genotype and allele frequencies of the SNPs of *DKK1* in Chinese Merino, Table S4: Haplotype frequencies of *DKK1* in Chinese Merino, Table S5: Genetic (below diagonal) and phenotypic correlation (above diagonal) coefficient between wool production and quality traits, Table S6: A summary of the raw data (phenotypes), Table S7: the least square means of the line effects of SNP in *DKK1*, Table S8: The allele substitution effect of *DKK1* on wool production and quality traits in Chinese Merino, Figure S1: The corresponding quantile–quantile (Q–Q) plots for the association of SNPs of *DKK1*.
