# Supplementary material for: Structural Characterization and Association of Ovine Dickkopf-1 Gene with Wool Production and Quality Traits in Chinese Merino
Source: Genes (Basel). 2017 Dec 20;8(12):400. doi: 10.3390/genes8120400 (PMC5748718; doi:10.3390/genes8120400)
Supplement: Supplementary file 1 [file genes-08-00400-s001.pdf]

Table S1. Information for *DKK1* in 16 different animal species

Species	Genbank mRNA accession number	Location	Genbank protein Accession number	Length[1] （AA）
African clawed frog	NM_001085592.1	chromosome="7S"	NP_001079061.1	259
Western clawed frog	NM_001016283.2	chromosome="7"	NP_001016283.1	257
Goat	XM_005698161.3	chromosome="26"	XP_005698218.1	262
Sheep	XM_012138945.2	chromosome="22"	XP_011994335.1	262
Cattle	NM_001205544.1	chromosome="26"	NP_001192473.1	265
Pig	NM_001145384.1	chromosome="14"	NP_001138856.1	266
Chimpanzee	XM_001163253.2	chromosome="10"	XP_001163253.1	266
Human	NM_012242.2	chromosome="10"	NP_036374.1	266
Rhesus monkey	XM_001098844.2	chromosome="9"	XP_001098844.1	266
Rabbit	NM_001082737.1	chromosome="18"	NP_001076206.1	268
House mouse	NM_010051.3	chromosome="19"	NP_034181.2	272
Norway rat	NM_001106350.1	chromosome="1"	NP_001099820.1	270
Dog	XM_846885.2	chromosome="26"	XP_851978.2	252
Zebrafish	AF116852.1	chromosome="12"	AAD22461.1	241
Chicken	XM_421563.2	chromosome="6"	XP_421563.2	338
Atlantic salmon	NM_001141456.1	chromosome="ssa19"	NP_001134928.1	169

Length[1] = DKK1 protein length (amino acids) in 16 different animals.

Table S2. The similarities of the deduced amino acid sequences of DKK1 protein in 16 different animal species

Species	2	3	4	5	6	7	8	9	10	11	12	13	14	15	16
African clawed frog	94	49	**49**	51	53	53	53	54	54	51	51	50	48	35	30
Western clawed frog	-	50	**50**	51	54	54	55	54	54	52	51	51	49	35	29
Goat	-	-	**98**	90	83	83	83	83	79	73	72	69	47	37	31
Sheep	-	-	-	**90**	**83**	**83**	**83**	**83**	**80**	**72**	**73**	**69**	**48**	**36**	**31**
Cattle	-	-	-	-	89	90	89	90	84	77	78	70	44	37	32
Pig	-	-	-	-	-	91	90	91	87	87	81	73	45	38	33
Chimpanzee	-	-	-	-	-	-	99	99	88	81	81	78	46	36	32
Human	-	-	-	-	-	-	-	98	87	81	81	70	46	36	32
Rhesus monkey	-	-	-	-	-	-	-	-	88	80	81	70	45	36	31
Rabbit	-	-	-	-	-	-	-	-	-	80	82	70	45	37	32
House mouse	-	-	-	-	-	-	-	-	-	-	93	67	45	38	30
Norway rat	-	-	-	-	-	-	-	-	-	-	-	67	45	38	31
Dog	-	-	-	-	-	-	-	-	-	-	-	-	44	34	33
Zebrafish	-	-	-	-	-	-	-	-	-	-	-	-	-	34	41
Chicken	-	-	-	-	-	-	-	-	-	-	-	-	-	-	25
Atlantic salmon	-	-	-	-	-	-	-	-	-	-	-	-	-	-	-

The numbers 2 to16 represent Western clawed frog, Goat, Sheep, Cattle, Pig, Chimpanzee, Human, Rhesus monkey, Rabbit, House mouse, Norway rat, Dog, Zebrafish, Chicken and Atlantic salmon, respectively.

Table S3. Genotype and allele frequencies of the SNPs of *DKK1* in Chinese Merino

SNPs	Line[2]	Numbers	Genotype frequency			Allele frequency		χ^2value (P value)[3]
			AA	AC	CC	A	C	
SNP2[1]	SF	179	0.050	0.318	0.631	0.209	0.791	χ^2=62.57 (P=3.58×10^{-12})
	PW	138	0.022	0.290	0.688	0.167	0.833	
	A	148	0.101	0.405	0.493	0.304	0.696	
	B	98	0.143	0.337	0.520	0.311	0.689	
	U	34	0.088	0.500	0.412	0.338	0.662	
	PM	128	0.008	0.141	0.852	0.078	0.922	
			II	ID	DD	I	D	
SNP3	SF	167	0.150	0.174	0.677	0.237	0.763	χ^2=62.49 (P=3.71×10^{-12})
	PW	129	0.070	0.202	0.729	0.171	0.829	
	A	136	0.184	0.235	0.581	0.301	0.699	
	B	91	0.242	0.176	0.582	0.330	0.670	
	U	33	0.333	0.212	0.455	0.439	0.561	
	PM	122	0.074	0.049	0.877	0.098	0.902	
			TT	TG	GG	T	G	
SNP5	SF	179	0.050	0.196	0.754	0.148	0.852	χ^2=62.14 (P=4.40×10^{-12})
	PW	136	0.022	0.147	0.831	0.096	0.904	
	A	149	0.101	0.228	0.671	0.215	0.785	
	B	103	0.136	0.291	0.573	0.282	0.718	
	U	34	0.059	0.353	0.588	0.235	0.765	
	PM	127	0.008	0.094	0.898	0.055	0.945	
			AA	AG	GG	A	G	
SNP6	SF	177	0.181	0.424	0.395	0.393	0.607	χ^2=44.37 (P=1.95×10^{-8})
	PW	136	0.125	0.544	0.331	0.397	0.603	
	A	147	0.211	0.476	0.313	0.449	0.551	
	B	98	0.173	0.582	0.245	0.464	0.536	
	U	36	0.250	0.389	0.361	0.444	0.556	

	PM	128	0.039	0.344	0.617	0.211	0.789	
			AA	AG	GG	A	G	
	SF	180	0.122	0.411	0.467	0.328	0.672	
	PW	138	0.087	0.514	0.399	0.344	0.656	
SNP7	A	148	0.128	0.432	0.439	0.345	0.654	χ^2=36.97
	B	102	0.176	0.559	0.265	0.456	0.544	(P=6.07×10^{-7})
	U	35	0.143	0.286	0.571	0.286	0.714	
	PM	130	0.023	0.346	0.631	0.196	0.804	
			CC	CT	TT	C	T	
	SF	137	0.007	0.292	0.701	0.153	0.847	
	PW	108	0.019	0.343	0.639	0.190	0.810	
SNP8	A	108	0.065	0.407	0.528	0.269	0.731	χ^2=22.36
	B	78	0.038	0.333	0.628	0.205	0.795	(P=4.48×10^{-4})
	U	29	0.172	0.448	0.379	0.397	0.603	
	PM	96	0.031	0.406	0.563	0.234	0.766	
			CC	CT	TT	C	T	
	SF	174	0.230	0.460	0.310	0.460	0.540	
	PW	132	0.205	0.606	0.189	0.508	0.492	
SNP9	A	142	0.338	0.444	0.218	0.560	0.440	χ^2=32.72
	B	89	0.416	0.449	0.135	0.640	0.360	(P=4.26×10^{-6})
	U	34	0.412	0.412	0.176	0.618	0.382	
	PM	127	0.165	0.480	0.354	0.406	0.594	
			TT	TC	CC	T	C	
	SF	169	0.207	0.284	0.509	0.349	0.651	
	PW	126	0.175	0.365	0.460	0.357	0.643	
SNP10	A	140	0.300	0.257	0.443	0.429	0.571	χ^2=75.93
	B	95	0.295	0.432	0.274	0.511	0.489	(P=5.95×10^{-15})
	U	30	0.300	0.200	0.500	0.400	0.600	
	PM	119	0.034	0.210	0.756	0.139	0.861	

			TT	TC	CC	T	C	
	SF	166	0.554	0.024	0.422	0.566	0.434	
	PW	128	0.523	0.070	0.406	0.559	0.441	
SNP11	A	141	0.525	0.057	0.418	0.553	0.447	χ^2=53.05
	B	98	0.602	0.092	0.306	0.648	0.352	(P=3.28×10^{-10})
	U	34	0.441	0.000	0.559	0.441	0.559	
	PM	108	0.287	0.074	0.639	0.324	0.676	

[1] SNP2 is good surrogates for SNP1 and SNP4, SNP2 is a haplotype tag SNP (ht SNP); [2] SF, PW, A, B, U and PM represent Super fine wool strain, Prolific wool strain, A strain, B strain, U strain and the Prolific meat strain, respectively; [3] The numbers shown in parentheses indicate the *P* value.

Table S4. Haplotype frequencies of *DKK1* in Chinese Merino

Haplotype Frequencies	Lines					
	A	B	PW	PM	U	SF
H1:AAICGAGCCCC	8.67%	3.96%	10.14%	4.72%	14.71%	7.82%
H2:AAICTAATCTT	19.33%	26.73%	5.80%	3.94%	26.47%	17.32%
H3:GCDGGAATCTT	18.00%	32.67%	36.96%	18.90%	11.76%	24.58%
H4:GCDGGGGCCCC	0.00%	3.96%	5.80%	23.62%	11.76%	1.68%
H5:GCDGGGGTTCC	24.00%	18.81%	21.74%	40.94%	17.65%	32.96%
H6:AAICGAGCCTT	6.00%	0.99%	3.62%	0.00%	5.88%	3.91%
H7:AAICTGGCCCC	6.00%	0.99%	3.62%	1.57%	5.88%	3.35%
H8:AAICTGGCCTC	7.33%	8.91%	5.07%	2.36%	2.94%	2.23%
H9:GCDGGAACCCT	7.33%	0.99%	5.80%	3.15%	2.94%	4.47%
H10:GCDGGAACCTT	2.67%	0.99%	0.72%	0.00%	0.00%	0.00%
H11:GCDGGGGTTCT	0.67%	0.00%	0.72%	0.79%	0.00%	1.68%
H12:GCIGGAATCTT	0.00%	0.99%	0.00%	0.00%	0.00%	0.00%

SF, PW, A, B, U and PM represent Super fine wool strain, Prolific wool strain, A strain, B strain, U strain and the Prolific meat strain, respectively.

Table S5. Genetic (below diagonal) and phenotypic (above diagonal) correlation coefficients between wool production and quality traits

	MFD	FDSD	CVFD	Curvature	FW	WFL
MFD		0.57±0.026**	-0.04±0.036	-0.41±0.030**	0.11±0.048*	0.05±0.037
FDSD	0.83±0.148**		0.78±0.013**	-0.41±0.031**	0.16±0.049**	0.02±0.037
CVFD	0.27±0.341**	0.73±0.130**		-0.21±0.035**	-0.11±0.019*	-0.01±0.037
Curvature	-0.79±0.151**	-0.79±0.187**	-0.43±0.290		-0.11±0.049**	-0.20±0.036**
FW	0.10±0.156**	0.04±0.175**	-0.30±0.083*	-0.17±0.130**		0.27±0.047**
WFL	0.13±0.171**	0.14±0.189**	0.27±0.274*	-0.20±0.135**	0.70±0.200**	

MFD, mean wool fiber diameter; FDSD, standard deviation of fiber diameter; CVFD, coefficient of variation of fiber diameter; WFL, wool fiber length; FW, fleece weight; * Significant $P<0.05$; ** Significant $P<0.01$; SF, PW, A, B, U and PM represent Super fine wool strain, Prolific wool strain, A strain, B strain, U strain and the Prolific meat strain, respectively.

Table S6. A summary of the phenotypic data

Traits	Lines	Mean	SD	range	CV	SE
MFD (μm)	A	21.10	1.806	10.200	3.262	0.147
	B	20.48	1.855	9.740	3.440	0.183
	PW	20.97	1.747	10.600	3.053	0.149
	PM	20.55	1.494	7.250	2.232	0.129
	U	21.26	2.026	10.960	4.105	0.338
	SF	19.03	1.736	10.200	3.014	0.129
FDSD (μm)	A	4.33	0.637	3.550	0.406	0.052
	B	4.19	0.577	2.860	0.333	0.057
	PW	4.16	0.646	3.940	0.418	0.055
	PM	4.20	0.641	3.540	0.411	0.055
	U	4.30	0.640	2.760	0.410	0.107
	SF	3.67	0.489	2.350	0.239	0.036
CVFD	A	20.60	2.495	14.000	6.225	0.203
	B	20.49	2.602	13.600	6.771	0.256
	PW	19.84	2.758	14.800	7.608	0.235
	PM	20.48	3.102	18.300	9.623	0.268
	U	20.26	2.520	8.700	6.349	0.420
	SF	19.24	2.255	16.700	5.084	0.168
Curvature (/2.5 cm)	A	11.28	1.621	11.000	2.629	0.132
	B	11.86	2.044	10.000	4.177	0.201
	PW	11.82	1.877	9.000	3.522	0.160
	PM	12.00	2.397	18.000	5.744	0.207
	U	11.47	1.934	9.000	3.742	0.322
	SF	14.94	2.134	9.000	4.552	0.159
FW (kg)	A	4.77	0.812	3.700	0.660	0.091
	B	5.01	0.814	3.400	0.663	0.115
	PW	4.33	0.778	4.300	0.605	0.097
	PM	3.48	0.800	3.700	0.640	0.094
	U	4.76	1.033	3.600	1.068	0.258
	SF	4.51	0.794	3.800	0.630	0.076
WFL (cm)	A	9.50	1.270	6.000	1.612	0.103
	B	9.50	1.270	6.000	1.614	0.125
	PW	9.42	1.308	6.500	1.710	0.111
	PM	8.70	1.244	7.000	1.547	0.107
	U	9.49	1.371	6.000	1.878	0.228
	SF	9.20	1.100	6.000	1.210	0.082

MFD, mean wool fiber diameter; FDSD, standard deviation of fiber diameter; CVFD, coefficient of variation of fiber diameter; WFL, wool fiber length; FW, fleece weight; SF, PW, A, B, U and PM represent Super fine wool strain, Prolific wool strain, A strain, B strain, U strain and the Prolific meat strain, respectively.

Table S7. The least square means of the line effects of *DKK1* SNPs in six Chinese Merino strains

	Traits	Lines					
		A	B	PW	PM	U	SF
SNP1	MFD (μm)	20.88±0.429^{ab}	20.12±0.525^{bc}	21.95±0.330^{a}	21.29±0.461^{ab}	22.46±0.732^{a}	19.25±0.540^{c}
	FDSD (μm)	4.37±0.143^{ab}	3.97±0.174^{bc}	4.39±0.110^{a}	4.57±0.153^{a}	4.39±0.244^{ab}	3.71±0.180^{c}
	CVFD	20.98±0.584	19.72±0.714	20.02±0.450	21.44±0.628	19.47±0.997	19.19±0.736
	Curvature (/2.5 cm)	11.42±0.444^{c}	13.21±0.542^{b}	11.25±0.342^{c}	11.36±0.476^{c}	11.84±0.757^{bc}	15.30±0.558^{a}
	FW (kg)	4.89±0.171^{a}	4.87±0.218^{ab}	4.24±0.143^{c}	3.58±0.189d	4.70±0.301^{abc}	4.31±0.225^{bc}
	WFL (cm)	9.06±0.258	9.15±0.316	8.66±0.199	8.18±0.277	9.03±0.441	9.45±0.325
		A	B	PW	PM	U	SF
SNP2	MFD (μm)	20.86±0.430^{b}	20.01±0.531^{bc}	21.95±0.332^{a}	20.51±0.600^{bc}	21.71±0.727^{ab}	19.25±0.541^{c}
	FDSD (μm)	4.37±0.143^{ab}	3.94±0.177^{bc}	4.39±0.110^{a}	4.40±0.200^{ab}	4.24±0.242^{abc}	3.70±0.180^{c}
	CVFD	21.00±0.588	19.69±0.727	20.02±0.454	21.45±0.822	19.45±0.995	19.16±0.740
	Curvature (/2.5 cm)	11.44±0.452^{c}	13.27±0.558^{b}	11.25±0.349^{c}	12.65±0.631^{bc}	11.59±0.764^{bc}	15.28±0.569^{a}
	FW (kg)	4.89±0.170^{a}	4.79±0.220^{ab}	4.24±0.143^{c}	3.31±0.245d	4.21±0.297^{bc}	4.34±0.224^{bc}
	WFL (cm)	9.05±0.258	9.08±0.318	8.66±0.199	8.06±0.360	8.48±0.436	9.46±0.324
		A	B	PW	PM	U	SF
SNP3	MFD(μm)	20.77±0.389^{b}	20.02±0.561^{bc}	21.41±0.493^{ab}	21.04±0.643^{ab}	22.30±0.587^{a}	18.91±0.357^{c}
	FDSD (μm)	4.30±0.136^{ab}	3.89±0.196^{bc}	4.31±0.172^{ab}	4.25±0.225^{ab}	4.46±0.205^{a}	3.60±0.125^{c}
	CVFD	20.76±0.575	19.41±0.830	20.10±0.731	20.18±0.952	20.02±0.869	19.02±0.529
	Curvature(/2.5 cm)	11.49±0.414^{c}	13.53±0.597^{b}	11.00±0.526^{c}	11.65±0.685^{c}	10.36±0.625^{c}	15.56±0.380^{a}
	FW(kg)	4.74±0.168^{a}	4.79±0.252^{ab}	4.14±0.216^{b}	3.10±0.278^{c}	4.21±0.254^{ab}	4.37±0.157^{ab}
	WFL(cm)	9.11±0.251	9.06±0.362	8.90±0.319	8.06±0.415	9.55±0.379	9.15±0.231
		A	B	PW	PM	U	SF
SNP4	MFD (μm)	21.06±0.409^{ab}	20.11±0.514^{bc}	21.96±0.329^{a}	21.05±0.523^{ab}	21.71±0.711^{ab}	19.24±0.529^{c}
	FDSD (μm)	4.35±0.137^{ab}	3.96±0.172^{bc}	4.36±0.110^{ab}	4.60±0.175^{a}	4.24±0.238^{abc}	3.70±0.177^{c}
	CVFD	20.69±0.566	19.69±0.713	19.88±0.455	21.87±0.724	19.45±0.985	19.16±0.733
	Curvature (/2.5 cm)	11.39±0.445^{c}	13.25±0.560^{b}	11.29±0.357^{c}	11.86±0.569^{bc}	11.60±0.773^{bc}	15.29±0.575^{a}
	FW (kg)	4.86±0.160^{a}	4.84±0.216^{ab}	4.22±0.145^{c}	3.38±0.217d	4.20±0.295^{bc}	4.33±0.222^{abc}
	WFL (cm)	9.18±0.251^{ab}	9.15±0.316^{ab}	8.67±0.202^{bc}	8.05±0.321^{c}	8.48±0.437^{abc}	9.46±0.325^{a}
		A	B	PW	PM	U	SF
SNP5	MFD (μm)	20.97±0.398^{ab}	20.17±0.519^{bc}	21.95±0.429^{a}	20.24±0.771^{abc}	21.80±0.809^{ab}	19.53±0.538^{c}
	FDSD (μm)	4.33±0.134^{a}	3.90±0.174^{bc}	4.27±0.144^{ab}	4.12±0.259^{abc}	4.54±0.272^{ab}	3.75±0.181^{c}
	CVFD	20.69±0.543	19.34±0.709	19.48±0.587	20.29±1.053	20.94±1.105	19.17±0.735
	Curvature (/2.5 cm)	11.43±0.442^{c}	13.04±0.576^{b}	11.66±0.477^{bc}	12.83±0.856^{bc}	11.01±0.898^{bc}	15.28±0.598^{a}
	FW (kg)	4.81±0.164^{a}	4.66±0.224^{a}	4.39±0.193^{a}	2.90±0.327^{b}	4.76±0.343^{a}	4.29±0.233^{a}
	WFL (cm)	9.22±0.251^{a}	8.91±0.327^{ab}	8.39±0.271^{b}	7.91±0.486^{b}	9.96±0.510^{a}	9.50±0.339^{a}
		A	B	PW	PM	SF	U
SNP6	MFD(μm)	21.19±0.358^{a}	21.08±0.465^{a}	21.95±0.465^{a}	20.84±0.692^{a}	18.96±0.310^{b}	NE
	FDSD (μm)	4.20±0.155^{a}	4.21±0.155^{a}	4.21±0.231^{a}	3.67±0.104^{a}	20.44±0.471^{b}	NE
	CVFD	19.15±0.611	20.10±0.910	19.30±0.408	11.56±0.387	11.85±0.502	NE

	Curvature (/2.5 cm)	12.10±0.747^{b}	15.31±0.335^{b}	4.83±0.140^{b}	4.88±0.221^{b}	4.47±0.197^{a}	NE
	FW (kg)	4.49±0.133^{a}	9.34±0.230^{a}	9.57±0.298^{a}	9.26±0.298^{b}	7.82±0.444^{a}	NE
	WFL (cm)	9.22±0.251^{a}	8.91±0.327^{a}	8.39±0.271^{a}	7.91±0.486^{b}	9.96±0.510^{a}	NE
SNP7		A	B	PW	PM	U	SF
	MFD (μm)	20.99±0.400^{ab}	20.51±0.511^{b}	21.79±0.500^{ab}	20.53±0.583^{b}	22.39±0.695^{a}	18.53±0.355^{c}
	FDSD (μm)	4.30±0.133^{a}	4.16±0.170^{a}	4.18±0.166^{a}	4.44±0.194^{a}	4.42±0.231^{a}	3.63±0.118^{b}
	CVFD	20.51±0.541	20.25±0.690	19.21±0.675	21.63±0.788	19.72±0.939	19.59±0.480
	Curvature (/2.5 cm)	11.49±0.432^{b}	12.13±0.552^{b}	10.90±0.540^{b}	12.24±0.630^{b}	11.29±0.751^{b}	15.39±0.384^{a}
	FW (kg)	4.75±0.159^{a}	4.80±0.257^{a}	4.12±0.208^{b}	3.23±0.238^{c}	4.61±0.284^{ab}	4.46±0.146^{ab}
	WFL (cm)	9.14±0.242	9.44±0.309	9.17±0.302	8.08±0.353	9.12±0.420	8.90±0.215
SNP8		A	B	PW	PM	U	SF
	MFD(μm)	20.33±0.549^{b}	20.34±0.499^{b}	21.99±0.495^{a}	21.11±0.477^{ab}	21.14±0.741^{ab}	19.02±0.347^{c}
	FDSD (μm)	4.30±0.177^{a}	4.17±0.161^{a}	4.37±0.160^{a}	4.39±0.154^{a}	4.19±0.239^{a}	3.61±0.112^{b}
	CVFD	21.18±0.727	20.61±0.661	19.86±0.656	20.79±0.632	19.79±0.981	18.95±0.460
	Curvature (/2.5 cm)	11.71±0.570^{b}	11.96±0.518^{b}	11.45±0.514^{b}	10.96±0.495^{b}	11.97±0.769^{b}	15.27±0.360^{a}
	FW (kg)	4.95±0.201^{a}	4.95±0.195^{a}	4.59±0.191^{ab}	3.72±0.182^{c}	4.13±0.282^{bc}	4.36±0.135^{b}
	WFL (cm)	9.24±0.333^{ab}	9.50±0.303^{a}	8.23±0.300^{c}	8.26±0.289^{c}	8.44±0.449^{bc}	9.06±0.210^{ab}
SNP9		A	B	PW	PM	U	SF
	MFD (μm)	20.64±0.349^{ab}	20.37±0.412^{b}	21.62±0.381^{a}	21.01±0.341^{ab}	20.60±0.821^{ab}	18.68±0.304^{c}
	FDSD (μm)	4.32±0.124^{a}	4.02±0.146^{a}	4.34±0.135^{a}	4.37±0.121^{a}	3.95±0.291^{ab}	3.66±0.108^{b}
	CVFD	20.98±0.514	19.81±0.607	20.11±0.561	20.76±0.502	19.36±1.208	19.54±0.447
	Curvature (/2.5 cm)	11.40±0.396^{c}	12.71±0.468^{b}	11.14±0.432^{c}	11.71±0.387^{bc}	13.55±0.931^{ab}	15.17±0.344^{a}
	FW (kg)	4.86±0.151^{a}	4.91±0.188^{a}	4.06±0.188^{b}	3.46±0.152^{c}	4.63±0.366^{ab}	4.43±0.137^{b}
	WFL (cm)	9.38±0.226^{a}	9.36±0.268^{a}	8.95±0.247^{a}	8.12±0.221^{b}	8.96±0.532^{ab}	9.05±0.197^{a}
SNP10		A	B	PW	PM	SF	U
	MFD (μm)	21.04±0.339^{b}	20.52±0.382^{b}	22.36±0.437^{a}	21.22±0.485^{ab}	18.91±0.301^{c}	NE
	FDSD (μm)	4.06±0.120^{a}	4.34±0.138^{a}	4.07±0.153^{a}	3.65±0.095^{a}	20.53±0.432^{b}	NE
	CVFD	19.38±0.558	19.14±0.620	19.28±0.385	11.52±0.363	12.28±0.410	NE
	Curvature (/2.5 cm)	11.65±0.521^{bc}	15.41±0.324^{b}	4.78±0.129^{c}	4.94±0.158^{bc}	4.50±0.188^{a}	NE
	FW (kg)	4.58±0.124^{a}	9.25±0.220^{a}	9.37±0.248^{a}	9.19±0.284^{b}	8.18±0.315^{a}	NE
	WFL (cm)	9.24±0.333^{a}	9.50±0.303^{a}	8.23±0.300^{a}	8.26±0.289^{b}	8.44±0.449^{a}	NE
SNP11		A	B	PW	PM	U	SF
	MFD (μm)	20.92±0.444^{ab}	20.16±0.497^{bc}	22.03±0.378^{a}	21.08±0.457^{ab}	22.39±0.698^{a}	19.59±0.466^{c}
	FDSD (μm)	4.39±0.142^{a}	4.04±0.159^{ab}	4.33±0.121^{a}	4.22±0.147^{a}	4.42±0.224^{a}	3.79±0.150^{b}
	CVFD	20.97±0.591	20.08±0.662	19.69±0.504	20.05±0.608	19.72±0.930	19.30±0.621
	Curvature (/2.5 cm)	11.30±0.441^{b}	12.18±0.493^{b}	11.08±0.376^{b}	11.78±0.454^{b}	11.30±0.693^{b}	14.65±0.463^{a}
	FW (kg)	4.82±0.165^{ab}	5.04±0.201^{a}	4.07±0.162^{c}	3.40±0.179d	4.61±0.274^{abc}	4.36±0.184^{bc}
	WFL (cm)	9.24±0.263	9.27±0.295	8.65±0.225	8.28±0.271	9.13±0.414	9.02±0.277

a, b,c Means within a row with no common superscript are different (*P*<0.05); MFD, mean wool fiber diameter; FDSD, standard deviation of fiber diameter; CVFD, coefficient of variation of fiber diameter; WFL, wool fiber length; FW, fleece weight; SF, PW, A, B, U and PM represent Super fine wool strain, Prolific wool strain, A strain, B strain, U strain and the Prolific meat strain, respectively; NE stands for not estimable.

Table S8. The allele substitution effects of *DKK1* SNPs on wool production and quality traits in Chinese Merino

	Traits[1]	A	B	PW	PM	U	SF
SNP1	MFD (μm)	0.00	-1.08	NE	NE	NE	0.76
	FDSD (μm)	0.08	-0.19	NE	NE	NE	0.19
	CVFD	0.42	-0.11	NE	NE	NE	0.14
	Curvature (/2.5 cm)	-0.02	3.96	NE	NE	NE	0.14
	FW (kg)	0.20	-0.31	NE	NE	NE	-0.22
	WFL (cm)	-0.23	-0.54	NE	NE	NE	0.98
SNP2	MFD (μm)	0.11	-1.16	NE	NE	NE	0.75
	FDSD (μm)	0.07	-0.25	NE	NE	NE	0.09
	CVFD	0.25	-0.25	NE	NE	NE	-0.38
	Curvature (/2.5 cm)	-0.07	3.91	NE	NE	NE	0.20
	FW (kg)	0.18	-0.38	NE	NE	NE	-0.28
	WFL (cm)	-0.19	-0.90	NE	NE	NE	0.94
SNP3	MFD (μm)	0.08	-0.38	-1.44	-0.28	NE	-0.96
	FDSD(μm)	0.00	-0.25	-0.42	0.29	NE	-0.07
	CVFD	-0.02	0.10	-0.68	1.59	NE	0.55
	Curvature(/2.5 cm)	-0.22	3.72	-0.92	0.84	NE	0.85
	FW(kg)	0.04	-0.34	-0.47	0.81	NE	-0.17
	WFL (cm)	-0.09	-0.89	0.98	0.95	NE	0.36
SNP4	MFD (μm)	0.27	-0.91	NE	NE	NE	0.69
	FDSD (μm)	0.18	-0.18	NE	NE	NE	0.12
	CVFD	0.61	-0.14	NE	NE	NE	-0.13
	Curvature (/2.5 cm)	-0.17	3.21	NE	NE	NE	0.20
	FW (kg)	0.26	-0.28	NE	NE	NE	-0.20
	WFL (cm)	-0.29	-0.57	NE	NE	NE	0.94
SNP5	MFD (μm)	-0.24	0.11	NE	NE	NE	-0.45
	FDSD (μm)	-0.14	0.06	NE	NE	NE	-0.09
	CVFD	-0.48	0.38	NE	NE	NE	0.08
	Curvature (/2.5 cm)	0.13	-3.32	NE	NE	NE	-0.23
	FW (kg)	-0.32	0.42	NE	NE	NE	0.15
	WFL (cm)	0.46	0.16	NE	NE	NE	-0.97
SNP6	MFD (μm)	-0.12	-0.13	-0.49	-0.53	NE	-1.02
	FDSD (μm)	-0.25	0.22	-0.48	-0.28	NE	-0.19
	CVFD	-1.03	0.42	-1.84	-0.91	NE	0.03
	Curvature (/2.5 cm)	0.16	-0.70	-1.27	0.86	NE	0.63
	FW (kg)	-0.21	-0.07	-0.02	-0.67	NE	-0.18
	WFL (cm)	-0.04	-0.51	0.64	-0.47	NE	-0.10
SNP7	MFD (μm)	0.21	0.37	-0.21	-0.46	NE	-1.25

	FDSD (μm)	-0.23	0.13	-0.49	-0.13	NE	-0.15
	CVFD	-1.28	0.02	-2.07	-0.24	NE	0.56
	Curvature (/2.5 cm)	0.16	-0.23	-0.94	0.79	NE	0.69
	FW (kg)	-0.39	-0.29	-0.23	-0.43	NE	-0.09
	WFL (cm)	0.10	-0.46	0.86	0.06	NE	-0.49
SNP8	MFD (μm)	-2.39	NE	0.56	-0.52	NE	NE
	FDSD (μm)	-0.18	NE	0.21	0.00	NE	NE
	CVFD	1.44	NE	0.44	0.53	NE	NE
	Curvature (/2.5 cm)	0.75	NE	0.25	0.02	NE	NE
	FW (kg)	0.50	NE	-0.03	0.49	NE	NE
	WFL (cm)	0.06	NE	-0.85	0.01	NE	NE
SNP9	MFD (μm)	0.18	-0.03	0.18	0.20	2.36	-0.26
	FDSD (μm)	-0.09	-0.07	-0.24	0.12	0.59	-0.07
	CVFD	-0.64	-0.25	-1.30	0.36	0.35	-0.12
	Curvature (/2.5 cm)	-0.03	-0.73	-0.58	0.12	-3.75	0.33
	FW (kg)	-0.29	-0.21	0.00	0.16	-0.13	-0.22
	WFL (cm)	-0.17	-0.52	-0.29	0.48	0.41	-0.18
SNP10	MFD(μm)	0.15	0.46	-0.28	NE	NE	0.42
	FDSD(μm)	0.23	0.03	0.24	NE	NE	0.06
	CVFD	0.94	-0.35	1.34	NE	NE	-0.09
	Curvature(/2.5 cm)	-0.22	-0.50	0.95	NE	NE	-0.13
	FW(kg)	0.15	0.16	0.29	NE	NE	0.22
	WFL (cm)	-0.10	0.69	-0.40	NE	NE	0.17
SNP11	MFD (μm)	0.33	-0.81	0.46	0.19	NE	0.16
	FDSD (μm)	0.24	-0.05	0.33	0.07	NE	0.01
	CVFD	0.78	-1.03	1.11	0.16	NE	-0.09
	Curvature (/2.5 cm)	0.03	0.70	-0.13	0.20	NE	-0.29
	FW (kg)	0.21	0.15	0.03	0.31	NE	0.02
	WFL (cm)	-0.15	-0.31	0.27	0.05	NE	0.04

MFD, mean wool fiber diameter; FDSD, standard deviation of fiber diameter; CVFD, coefficient of variation of fiber diameter; WFL, wool fiber length; FW, fleece weight; SF, PW, A, B, U and PM represent Super fine wool strain, Prolific wool strain, A strain, B strain, U strain and the Prolific meat strain, respectively; NE stands for not estimable.

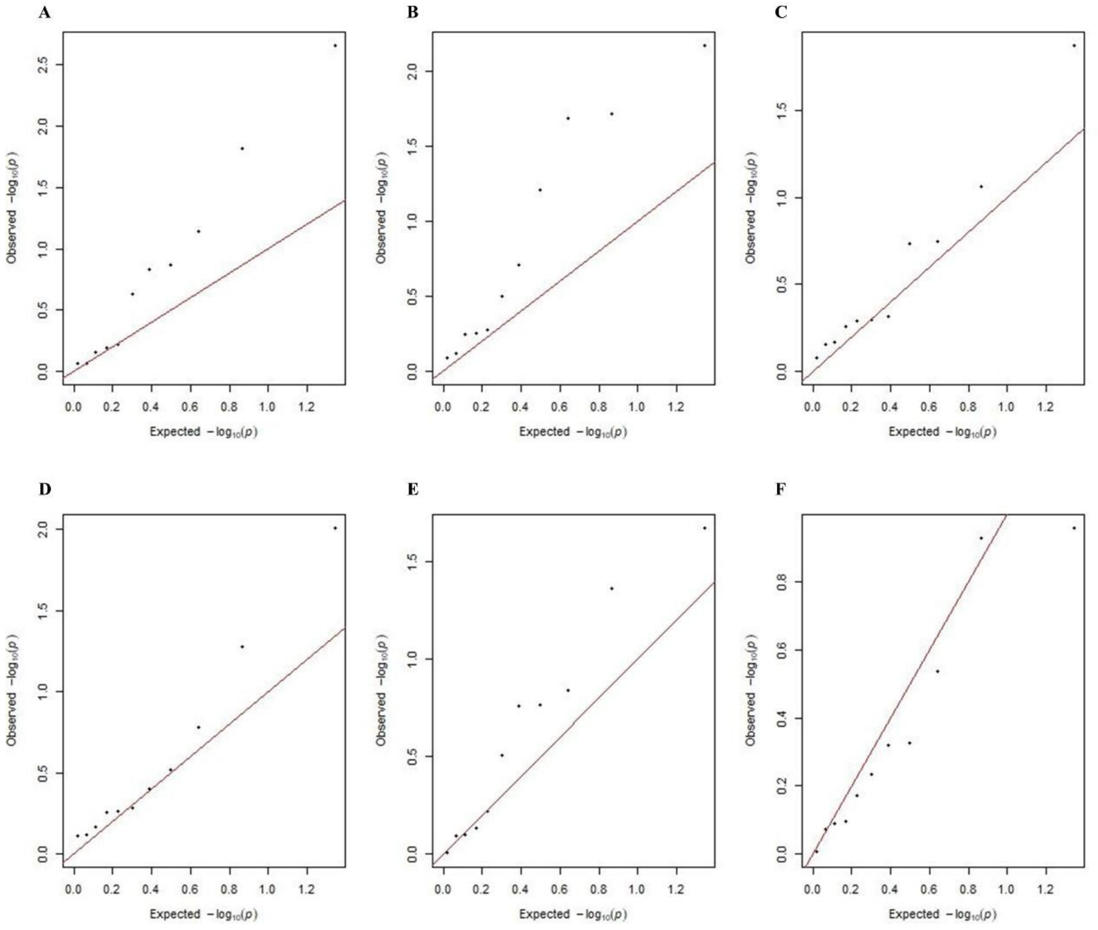

Figure S1. The corresponding quantile–quantile (Q-Q) plots for the association p values of *DKK1* SNPs. The red line shows the expected distribution of P-values; the black line shows the observed distribution of P-values. Fig.S1-A, S1-B, S1-C, S1-D, S1-E, S1-F refer to plot for MFD, FDSD, CVFD, Curvature, FW, and WFL respectively.
